# Diagnosis of Vertical Root Fracture with Cone-Beam Computerized Tomography in Endodontically Treated Teeth: Three Case Reports

**Published:** 2013-05-01

**Authors:** Daniela Cristina Miyagaki, Jefferson Marion, Caio Cézar Randi Ferraz

**Affiliations:** 1 Department of Restorative Dentistry, Piracicaba Dental School, State University of Campinas, UNICAMP, Piracicaba, Sao Paulo; 2 Department of Reparative Dentistry, Faculty of Dentistry, University of Maringá, UNINGA, Maringá, Paraná, Brazil

**Keywords:** Cone-Beam Computed Tomography, Dental Radiography, Diagnosis, Endodontics, Tooth Fractures

## Abstract

A definitive diagnosis of vertical root fracture (VRF) is often a challenging task for clinicians. This is because two dimensional periapical radiographs are usually unable to detect the fracture line due to the direction of the X-ray beam. This report presents a set of 3 cases of endodontically treated teeth that were diagnosed with VRFs based on findings from clinical, radiographic, and cone-beam computerized tomographic (CBCT) examinations. After extraction, VRFs were confirmed in all cases. The presence of periodontal pockets or other signs which would compromise the correct diagnosis could not be detected in all three cases. Fracture lines were only visible with the aid of CBCT which provided useful information for the diagnosis and management of VRF. However, the clinical and radiographic data should not be discarded, but used in conjunction with CBCT.

## 1. Introduction

Vertical root fracture (VRF) is the most severe type of longitudinal defect (1). It manifests as a complete or incomplete fracture line which extends obliquely or longitudinally through the enamel and dentin of an endodontically treated root (2). According to previous studies, longitudinal root fractures have a prevalence ranging from 3.7% to 30.8% for endodontically treated teeth (3, 4), and relatively uncommon in teeth without endodontic treatment (5). The causes may be related to eccentric forces from occlusion, trauma, excessive pressure during endodontic treatment, successive tooth restorations, poorly designed posts, or inappropriate tooth selection as a bridge abutment (6-8).

Detection of VRF is a significant challenge because clinical symptoms and radiographic signs are not completely pathognomonic. Generally, clinical signs, radiographic features and symptoms observed in VRFs are similar to those in a failed root canal treatment and periodontal disease, complicating the accomplishment of an accurate diagnosis (9). According to Hassan *et al*., it often requires prediction rather than definitive identification (10). Thus, the location and size of the defect cannot always be objectively assessed without extraction or simultaneous mucoperiosteal flap surgery (2).

VRFs usually have a poor prognosis and the selection of an appropriate treatment can be confusing for most clinicians. In a multi-rooted tooth compromised with VRF a more conservative treatment can be performed by resecting the involved root or by performing a new alternative surgical treatment with composite resin and a synthetic hydroxyapatite graft (11). Conversely, a single-rooted teeth usually have a poor prognosis, leading to extraction in 11-20% of cases (3). As the prognosis of root fractures worsens with time, and in order to avoid rapid bone loss and periodontal destruction (12), an early accurate diagnosis is essential to determine the most appropriate treatment technique.

VRF detection on conventional periapical radiographs is often challenging even if taken from different angles (13), especially when displacement of the fragments has not yet occurred as a result of granulation tissue and edema (14). Thus, the superimposition of other structures also limits their sensitivity for detecting longitudinal fractures (15). These problems may be overcome with alternative imaging systems such as cone beam computed tomography (CBCT) (16-18), which provides a 3-dimensional scan of the patient’s head (10). Recent studies have found that CBCT is more accurate than conventional periapical radiography for the detection of VRFs, as they allow direct visualization of the fracture lines (10, 19-21).

Considering that the use of such technology to diagnose these complications could be essential for an appropriate treatment planning (22), the present report describes 3 cases in which the diagnosis of VRF were made through the patient’s dental histories as well as clinical, radiographic and CBCT findings.

## 2. Case Reports

The patients were referred to a private office of endodontic clinic in Maringá, Brazil. The operating parameters for CBCT i-CAT® examination (Imaging Science International, Hatfield, PA, USA) were the following: 13 cm acquisition field, 40 s of acquisition duration, 0.25 mm of voxel, 120 kV, 46.72 mA. Tomographic sections of 1 mm in 3 planes (axial, coronal and sagital) were made. Each tooth was submitted to standardized periapical radiographic examination (Dabi Atlante 1070X equipment; Dabi Atlante, São Paulo, Brazil) with an exposure time of 0.7 s, 70 kV and 10 mA.

### 2.1. Case 1

**Figure 1.**
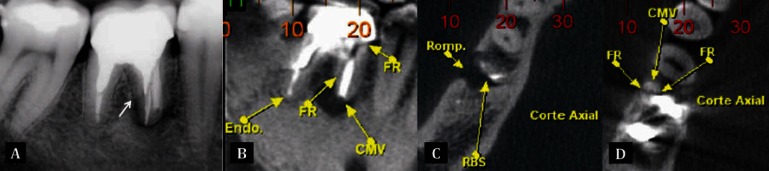
A) Radiograph of tooth 46 showing a possible line of fracture (white arrow); B,C,D) CBCT images in the sagital and axial planes of the tooth clearly shows fracture line (FR) and resorption area (RBS)

A 40-year-old man, without systemic involvement, complained of pain on pressure and had painful response to both vertical and horizontal percussion of his mandibular first right molar. Clinically, no periodontal pockets were found, nor swelling or pain on periapical palpation. Root canal therapy, as well as core and crown treatment was performed fifteen years ago. Radiographic examination revealed a lack of adaptation of the prosthesis, and a possible crack at the level of the post of the mesial root (Figure 1A). The CBCT scan revealed radiolucent lesion larger than the one visible on radiograph as can be seen in the axial section, which shows clearly an image compatible with vertical root fracture in booth sagittal and axial slices (Figures 1B, 1C, 1D). The tooth in question was extracted and replaced with an implant.

### 2.2. Case 2

**Figure 2.**
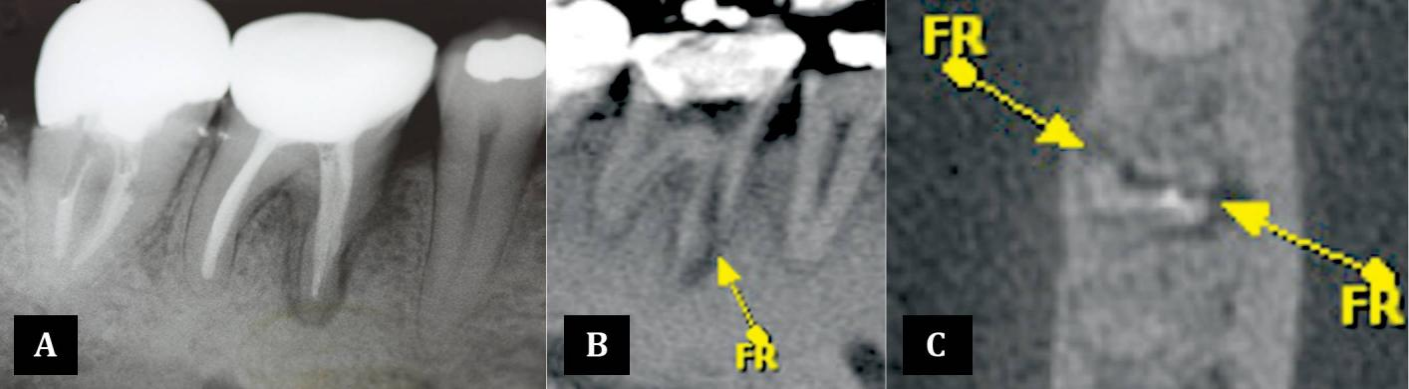
A) Periapical radiograph of tooth 46, showing radiolucent area on mesial side of the root (white arrow); B,C) CBCT images in the sagital and axial planes of the tooth, showing clearly the root fracture (FR) with separation of the fragment

A 52-year-old man with hypertension and diabetes reported spontaneous pain in the mandibular first right molar. The pain was characterized as short-term, intermittent and localized. An intraoral examination showed that it was sensitive to periapical palpation and percussion (horizontal and vertical) tests, but with no signs of edema or periodontal pocketing. Radiographic examination (05/21/10) revealed a radiolucent lesion in the mesial root (Figure 2A) and lack of cervical adaptation of the prosthesis. Therefore, the prosthesis and endodontic filling material were removed, and inserted an intracanal medication with calcium hydroxide associated with propylene glycol. After two weeks, the patient returns with sensitivity; a CBCT was hence forth recommended. The CBCT scans showed the presence of very legible vertical root fracture with displacement of the buccal-lingual fragment, as shown in both sagittal and axial slices (Figures 2B, 2C). The tooth was extracted and replaced with an implant.

### 2.3. Case 3

**Figure 3.**
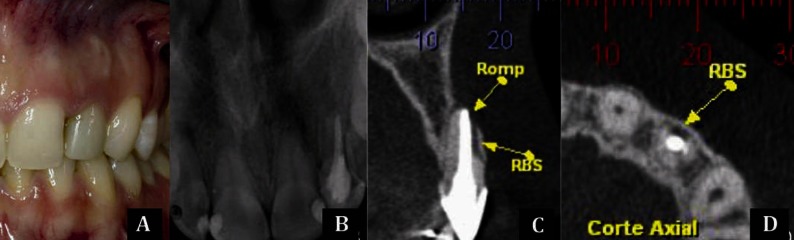
A) Clinical aspect of tooth 22; B) Periapical radiograph of anterior teeth showing no evidence of root fracture; C,D) CBCT images in the sagital and axial planes of the tooth showing an evident root fracture (FR) with separation of the fragment

A healthy 26 year-old patient, attended the endodontic office for treatment of maxillary right lateral incisor (teeth #12) and evaluation of maxillary left lateral incisor (teeth #22) due to probable root fracture found in CBCT by the orthodontist (Figure 3A). In the periapical radiograph (Figure 3B), despite a slight foreshortening, radiolucent areas involving the apices of teeth 12 and 21 were apparent; however, on tooth 22 no fracture line was noted, as previously indicated by CBCT. Clinically there was no edema, fistula, mobility or pain symptoms, and percussion test was negative. In addition, there was no history of trauma. The endodontist requested a new CBCT scan of tooth #22 to specifically observe the sagittal and axial slices, as the previous CBCT consisted of only one sagittal section. CBCT slices, both sagittal and axial planes, showed clearly the vertical root fracture with separation of the fragments and an area of resorption (Figures 3C, 3D). Therefore, extraction was indicated and performed.

## 3. Discussion

This new imaging technique is essential to verify the extension and exact location of the fracture and therefore indicate the most appropriate treatment In case 1, though a possible fracture line had been seen in the radiographic examination, it was only confirmed with the CBCT images. Extraction was performed since a resorption area and separation of the fragment was observed, which contraindicates any conservative treatment. The accomplishment of an early and accurate diagnosis avoided the occurrence of a possible bone loss, pain, and malfunction (23). Additionally, surgical procedures were no longer necessary in order to complement the diagnosis with CBCT imaging. Previously, the location and size of the defect could only be objectively assessed with extraction or simultaneous mucoperiosteal flap surgery (2).

In case 2, as the initial radiographic examination were not able to show the fracture line, and only indicated a radiolucent area, a CBCT examination was requested. Initially, the clinical signs suggested a diagnosis of acute chronic periapical abscess, however, the pain persistence led to another possible diagnosis: root fracture. The CBCT slices clearly demonstrated the fracture line with separation of the fragments and therefore extraction was indicated. In situations where patients have poorly localized odontogenic pains associated with an untreated or previously root treated tooth, CBCT may reveal the presence of previously undiagnosed pathosis (24, 25). And this knowledge is extremely important when diagnosing and managing a failing endodontic treatment (26).

Case 3 shows the importance of requesting the appropriate reconstruction plane (axial, sagittal or coronal) to observe the fracture line, especially when it is mesiodistally oriented (27). As previously reported by Hassan *et al.*, axial slices are more accurate than coronal and sagittal slices in detecting VRF (20). The diagnosis and treatment plan seemed to be complicated because no signs or symptoms were present. As a separation of the fragments could be seen with resorption of the area, conservative treatments were not indicated. Extraction was therefore performed. In this case and in case 1, CBCT diagnosis allowed the operators to prevent addition bone loss and avoid performing unnecessary diagnostic surgical procedures.

The clinical and radiographic diagnosis of VRFs is often complicated. A local deep pocket, dual sinus tracts, and a halo type of lateral radiolucency are among the symptoms (20). However, in all three cases no related symptoms were found, which complicated making a diagnosis. The exact diagnosis of a VRF is crucial to avoid erroneous extraction. Among the methods available to detect VRFs are digital imaging (28), illumination, x-rays, periodontal probing, staining, surgical exploration, bite tests, direct visualization of the fracture, operative-microscope examination, and CBCT scanning (29), the latter has been accepted as an innovative diagnostic tool.

As previously reported, dental CBCT and i-CAT, which was used in these cases, has shown to be more accurate in detecting VRF than conventional two-dimensional intraoral techniques, (1, 20). Until now, only few studies have used CBCT as a gold standard to accurately diagnose VRFs in endodontically treated teeth (30, 31). In all cases, VRFs diagnoses were based on CBCT findings, where the fracture lines were seen in sagittal and axial sections. Recently, Edlund *et al.* demonstrated the effectiveness of CBCT in detecting VRF, showing high sensitivity (88%) and specificity (75%) (32). However, image artifacts can occur due to the presence of radiopaque materials, such as metals, gutta-percha and sealers, which can mimic a fracture line (32-34). These artifacts did not interfere in the diagnosis of the three cases presented. When prognostic and diagnostic assessments still remains questionable, there is no definite substitute for direct visualization (6). Clinicians must be prepared to interpret not only the teeth involved but the entire CBCT image volume (35). This present report demonstrates the importance of using the available innovative technologies, such as CBCT, in endodontics practice alongside clinical signs and symptoms for diagnostic confirmation.

However, factors like high radiation dose, high cost, and lack of availability might preclude the use of CBCT in some cases (1). A judgment must be made individually and CBCT should only be considered in situations where information from conventional imaging does not yield adequate information to allow appropriate management of the endodontic problem (36).

## 4. Conclusion

This case reports emphasizes that the diagnosis of VRF in endodontically treated teeth is complex and CBCT images could be considered a reliable diagnostic method. However, clinical signs and symptoms and conventional radiographic evaluation are also fundamental to diagnosis.
